# Dietary Supplementation With CoQ10 and Sel‐Plex Mitigates the Effects of Elevated Water Temperature on Performance, Digestive Physiology, Immune Competence, and Antioxidant Balance in Rainbow Trout

**DOI:** 10.1155/anu/3450813

**Published:** 2026-04-30

**Authors:** Mohammad Hossein Dadfar, Ahmad Imani, Arya Vazirzadeh, Kourosh Sarvi Moghanlou, Faezeh Jamali

**Affiliations:** ^1^ Department of Fisheries, Faculty of Natural Resources, Urmia University, Urmia, Iran, urmia.ac.ir; ^2^ Department of Natural Resources and Environmental Engineering, School of Agriculture, Shiraz University, Shiraz, Iran, shirazu.ac.ir

**Keywords:** antioxidant, aquaculture nutrition, coenzyme Q10, immune competence, oxidative stress, rainbow trout, selenium, temperature

## Abstract

**Background/Aims:**

Elevated water temperatures can impair fish growth, immunity, and physiological performance. This study aimed to evaluate the effects of dietary coenzyme Q10 (CoQ10) and organic selenium (O‐Se; Sel‐Plex) supplementation on growth, digestive enzymes, innate immunity, antioxidant capacity, and the expression of stress‐ and immune‐related genes (*hsp70-α* and *il-1β*) in the liver of rainbow trout (*Oncorhynchus mykiss*) under thermal stress, measured using real‐time quantitative PCR (RT‐qPCR) with β‐actin as the reference gene.

**Methods:**

A total of 225 rainbow trout (21 ± 0.5 g) were stocked in 150‐L recirculating tanks at either optimal (15°C) or elevated (20°C) water temperatures. Fish were fed diets supplemented with CoQ10 (0 or 20 mg/kg) and/or Sel‐Plex (0 or 5 mg/kg) for 60 days. Growth indices, digestive enzyme activity, hepatic antioxidant capacity, immune parameters, and the expression of *hsp70-α* and *il-1β* were assessed.

**Results:**

Elevated temperature significantly reduced growth, feed efficiency, and immune status. Dietary supplementation with CoQ10 or O‐Se improved hepatic antioxidant capacity, digestive enzyme activity, and innate immunity, with the greatest effects observed when both supplements were combined. Cosupplementation also mitigated heat‐induced upregulation of HSP70‐α and IL‐1β and reduced liver malondialdehyde (MDA) levels.

**Conclusion:**

Combined dietary CoQ10 and O‐Se supplementation alleviate the adverse effects of elevated water temperatures on rainbow trout by enhancing growth, physiological performance, antioxidant defenses, and immune responses.

## 1. Introduction

Temperature has a pervasive and direct effect on the biochemical and physiological responses ofs poikilotherms (e.g., fish), whose body temperature varies with ambient conditions, leading to significant consequences for life history traits [1, 2]. In contrast, homeotherms (e.g., mammals and birds) maintain a relatively constant body temperature, making their physiological responses less directly dependent on environmental temperature, while heterotherms exhibit intermediate strategies [3]. Fish are considered indicators of climate change due to their natural critical thermal thresholds and limited ability to adjust thermal sensitivity to maintain homeostasis [4]. In this context, increases in ambient water temperature can disrupt fish physiology by altering metabolic rate, enzyme activity, feeding behavior, and immune function. Elevated temperatures also destabilize cellular biomolecules, including proteins, lipids, and nucleic acids, through protein denaturation, lipid peroxidation, and oxidative DNA damage [4]. By 2100, global mean surface air temperature (refers to the average temperature of the earth’s land and ocean surfaces) is projected to rise by 1–3.7°C [5], which is closely reflected in aquatic ecosystems, particularly in shallow or semi‐enclosed waters, due to enhanced heat transfer from the atmosphere [6]. Such thermal increases impose stress on aquatic organisms, reducing dissolved oxygen availability and influencing key physiological processes, including growth, immunity, and digestive enzyme activity [1].

The ideal rearing temperature refers to the temperature that promotes maximal growth and feeding performance while ensuring the overall health and well‐being of the fish. Environmental water temperature requirements have been determined for different salmonid species and are applied in commercial aquaculture operations, with optimal ranges of 12–18°C for rainbow trout, 10–16°C for Atlantic salmon (*Salmo salar*), 8–16°C for brown trout (*Salmo trutta*), and 4–12°C for Arctic char (*Salvelinus alpinus*), which are critical for maintaining growth, metabolism, and physiological performance [7]. In addition, the influence of controlled rearing temperatures on fish growth and physiological performance has been widely investigated [8–12].

Elevated water temperatures represent a major constraint in aquaculture, as they can disrupt digestive physiology, reduce enzymatic activity, and ultimately impair nutrient utilization and growth performance in fish [13, 14]. Thermal stress has been shown to accelerate intestinal transit, compromise epithelial integrity, and alter digestive secretions, thereby diminishing digestive efficiency and metabolic stability [15]. In response, producers commonly adopt adaptive management strategies such as feed restriction, enhanced aeration to maintain dissolved oxygen, and reduced stocking densities to alleviate heat‐induced stress [16]. Beyond husbandry adjustments, targeted nutritional interventions have emerged as sustainable and practical approaches to mitigate thermal stress. Modulation of dietary composition and functional supplementation can enhance physiological resilience under elevated temperatures. For example, increasing the dietary protein‐to‐energy ratio has improved growth and nutrient utilization in barramundi (*Lates calcarifer*) under thermal challenge [17]. Similarly, supplementation with mannanoligosaccharides in common carp [18], vitamin C in Wuchang bream (*Megalobrama amblycephala*) [19], and selenium nanoparticles (Se‐NPs) or omega‐3 fatty acids in striped catfish (*Pangasianodon hypophthalmus*) [4] has been reported to enhance antioxidant capacity, immune responses, and growth performance under elevated temperature conditions. Collectively, these findings underscore the importance of nutritional modulation as a complementary strategy to maintain physiological homeostasis in fish exposed to climate‐related thermal stress.

CoQ10, also known as ubiquinone, is a naturally occurring compound in biological systems that plays a crucial role in mitochondrial energy metabolism by shuttling electrons between complexes I/II and III in the electron transport chain (ETC), thereby facilitating ATP synthesis, and also contributes to the regeneration of the antioxidant α‐tocopherol by reducing its oxidized form [20]. It is well‐established as an effective lipid‐soluble antioxidant [21]. However, this compound is produced in insufficient quantities in the body, particularly during stressful conditions; therefore, it must be supplemented through feed. Moreover, studies have indicated that CoQ10 is a promising feed additive due to its efficient bioavailability and minimal toxicity in animals. Additionally, it may function as an antioxidant when included in aquaculture diets [22–24]. Se is an essential micronutrient necessary for the growth and physiological functions of aquatic animals, including fish [25, 26]. It is widely recognized that Se occurs in various forms, namely inorganic, organic, and nano‐sized, each exhibiting unique absorption mechanisms and metabolic pathways in animals [27]. Several studies have demonstrated that organic forms of SE (e.g., selenomethionine) are absorbed and utilized more efficiently by fish compared to inorganic forms (such as sodium selenite or selenate) [28–31]. This higher bioavailability of organic Se (O‐Se) results in improved incorporation into body tissues and enhanced physiological benefits, including antioxidant defense and growth performance.

Recent evidence suggests that dietary supplementation with functional feed additives, particularly antioxidants and their derivatives, can enhance immune competence and antioxidant defense mechanisms in fish subjected to environmental or biological stressors. For example, Se supplementation has been shown to improve oxidative status and stress tolerance in *P. hypophthalmus* exposed to combined lead and thermal stress [32]. Similarly, dietary coenzyme Q10 (CoQ10) and vitamin C enhanced resistance and immune‐related responses in Nile tilapia (*Oreochromis niloticus*) following bacterial challenge [33]. The inclusion of commercial additives containing plant extracts and prebiotics has also been reported to improve stress resilience in rainbow trout under elevated temperature conditions [34]. Furthermore, supplementation with oregano essential oil improved antioxidant capacity and physiological stability in *O. niloticus* during acute heat stress [35]. CoQ10 administration enhanced immune performance in European eel (*Anguilla anguilla*) challenged with pathogenic bacteria [24]. In addition, combined dietary supplementation with CoQ10, vitamin E, and Se‐NPs promoted antioxidant defenses and stress tolerance in rainbow trout subjected to air exposure [36]. Collectively, these findings support the role of antioxidant‐based nutritional strategies in reinforcing physiological homeostasis and improving stress adaptability in aquaculture species.

This study explores a novel aspect of aquaculture nutrition by examining the combined effects of dietary CoQ10 and O‐Se on rainbow trout exposed to elevated water temperatures, a condition of increasing relevance under climate change. Unlike previous studies that assessed these supplements individually, the present work evaluates their potential synergistic effects in mitigating heat‐induced stress. Multiple physiological and molecular endpoints were assessed, including growth performance, feed efficiency, hepatic antioxidant capacity, digestive enzyme activity, innate immunity, and the expression of stress‐ and immune‐related genes (*hsp70-α* and *il-1β*) in rainbow trout reared at either optimal (15°C) or elevated (20°C) temperatures. This integrative approach provides novel insights into antioxidant‐based nutritional strategies aimed at enhancing thermal tolerance and maintaining physiological homeostasis in aquaculture species under conditions of thermal stress.

## 2. Materials and Methods

### 2.1. Experimental Fish and Husbandry

A total of 720 healthy all‐female triploid juvenile rainbow trout (*Oncorhynchus mykiss*), averaging 21 ± 0.5 g in initial body weight, were obtained from a commercial aquaculture facility in Fars Province, Iran. The experiment was conducted in a controlled, laboratory‐simulated environment designed to replicate aquaculture conditions. After transfer, the fish experienced a 14‐day acclimation phase in 1000‐L polyethylene tanks. Upon completion of this period, the fish were assigned at random to 24 separate 500‐L tanks, maintaining a stocking density of 30 fish per tank. The experiment was structured as a completely randomized design, comprising eight experimental groups with three replicate tanks for each group. All tanks were integrated into a recirculating aquaculture system (RAS). The water temperature was thermostatically controlled to maintain two distinct thermal regimes: four groups (including 12 tanks) were kept at a normothermic condition of 15°C, while the other four were subjected to an elevated temperature of 20°C (as a climate change threshold for rainbow trout). Water dissolved oxygen and pH were monitored daily to ensure they remained within species‐specific optimal ranges. The averages of dissolved oxygen level and pH were 7.4 ± 0.3 mgL^−1^ and 7.6 ± 0.2, respectively. Throughout the 60‐day experimental period, the fish were manually fed to apparent satiation three times daily (08:00, 13:00, and 19:00).

### 2.2. Diet Formulation and Preparation

A basal diet was designed to satisfy the nutrient requirements of rainbow trout following the guidelines established by the [37]. The feedstuffs for the basal formulation were sourced from a local supplier (21 Beyza Feed Co., Fars Province, Iran). The composition and formulation details of this diet are summarized in Table 1. To prepare the feed, all dry components were first ground, accurately weighed, and thoroughly blended for 5 min to ensure homogeneity. The experimental diets (outlined in Table 2) were subsequently prepared by supplementing the basal mixture with specific antioxidants, including CoQ10 at 0 or 20 mg/kg feed and the O‐Se source Sel‐Plex at 0 or 5 mg/kg feed, as reported by Aramli et al. [36]. Distilled water was gradually incorporated to form a compact dough, which was then processed through a pelletizer to produce 3 mm pellets. Following preparation, the pellets were dried in an oven at 40°C for 24 h. The resulting diets were then sealed in airtight polyethylene bags and preserved at 4°C until their utilization in the feeding experiment.

**Table 1:** Formulation and proximate composition of the basal diet.

Ingredients	(%)	Proximate composition	(%)
Fish meal (kilka) Fish meal (sardine)	23.75 25	Moisture Protein	10 42
Fish oil Sunflower oil	7.5 7.5	Lipid Ash	16 9
Wheat flour	17.5	Carbohydrate	13
Soybean meal	10	Fiber	10
Wheat gluten	6.25	—	—
Methionine	0.75	—	—
Lysine	0.5	—	—
Soy lecithin	1.25	—	—
Total	100	—	—

**Table 2:** Experimental treatments implemented in the current research.

Diet no.	Nomenclature	*T* (°C)	O‐Se (mg/kg feed)	CoQ10 (mg/kg feed)
1 (Ctrl)	T_15_Se_0_Q_0_	15	0	0
2	T_15_Se_5_Q_0_	15	5	0
3	T_15_Se_0_Q_20_	15	0	20
4	T_15_Se_5_Q_20_	15	5	20
5	T_20_Se_0_Q_0_	20	0	0
6	T_20_Se_5_Q_0_	20	5	0
7	T_20_Se_0_Q_20_	20	0	20
8	T_20_Se_5_Q_20_	20	5	20

Abbreviations: CoQ10, coenzyme Q10; O‐Se, organic selenium.

### 2.3. Performance, Feed Utilization, and Carcass Contents

Upon completion of the 60‐day feeding trial, the fish in each replicate tank were enumerated and collectively weighed to evaluate growth metrics and feed utilization efficiency. Growth and nutrient efficiency indices including weight gain (WG), specific growth rate (SGR), daily growth rate (DGR), thermal growth coefficient (TGC), feed conversion ratio (FCR), protein efficiency ratio (PER), protein productive value, lipid efficiency ratio (LER), and lipid productive value were calculated according to Hoseyni et al. [38] and Noaman‐Hamad et al. [39].

Furthermore, to assess changes in nutrient deposition, fish samples were collected from the initial stock at the start of the trial and from each experimental tank at the end. These samples were subjected to whole‐body proximate composition analysis. Moisture, crude protein, crude lipid, and ash contents were analyzed in accordance with the methods established by the Association of Official Analytical Chemists [40].

### 2.4. Blood Sampling and Serum Biochemical Analysis

At the conclusion of the experimental trial, a representative subset of fish (e.g., three fish per tank) were sampled for serological analysis. Sampled fish were first anesthetized in a solution of clove powder (150 mg/L). Subsequently, blood samples were collected from the caudal vein using 2‐mL syringes without heparin. The samples were allowed to clot at ambient temperature for 1 h and subsequently centrifuged at 3000 × g for 15 min at 4°C to separate the serum. The resulting serum was carefully collected and used to measure key metabolic indices, including triglycerides (TGs), cholesterol (Chol), and glucose (Glu). Spectrophotometric analyses were conducted using commercially available diagnostic kits (Pars Azmoon, Tehran, Iran) following the protocols provided by the manufacturer [41].

### 2.5. Immunological Assays

Multiple immunoassays were performed to assess the intrinsic immune function of the fish under study.

#### 2.5.1. Serum Protein Analysis

Total protein concentration was determined spectrophotometrically using commercial diagnostic kits (Pars Azmoon, Tehran, Iran) based on the biuret reaction [42]. Serum albumin was measured using a standard colorimetric method employing bromocresol green [43]. Globulin concentration was subsequently calculated by subtracting albumin from total protein values for each individual sample.

#### 2.5.2. Lysozyme (Lyz) Assay

Serum Lyz levels were assessed using a turbidimetric assay in a microplate format, following the procedure outlined by Vazirzadeh et al. [44]. In brief, 10 µL of serum was combined with 90 µL of a *Micrococcus luteus* suspension (0.2 mg/mL in sodium phosphate buffer, pH 7.4). The decrease in absorbance at 450 nm was measured at the start and after 10 min using a microplate reader (BioTek, UK). Lyz activity was expressed in units, with one unit corresponding to a reduction in absorbance of 0.001 per minute.

#### 2.5.3. Respiratory Burst Activity (Nitroblue Tetrazolium [NBT] Assay)

The respiratory burst activity of blood phagocytes was assessed by quantifying the reduction of NBT, according to the protocol described by Vazirzadeh et al. [44]. A 50 µL aliquot of fresh blood was combined with 50 µL of 0.2% NBT solution and incubated for 30 min. Thereafter, 50 µL of this mixture was mixed with an equal volume of dimethylformamide. Following centrifugation, the absorbance of the formazan present in the supernatant was determined at 540 nm using a microplate reader (BioTek, UK).

#### 2.5.4. Total Immunoglobulin (Ig) Assay

Plasma Ig levels were measured via a PEG‐based precipitation method as described by Siwicki and Anderson [45]. This method involves measuring the total protein content of plasma samples both before and after the precipitation of Igs with a 12% PEG solution. The total plasma Ig level was calculated as the difference between these two protein measurements.

### 2.6. Liver Tissue Sampling and Antioxidant Status Assays

#### 2.6.1. Sample Collection and Preparation

Upon termination of the experiment, fish were euthanized through an overdose of clove powder anesthetic, followed by spinal transection. The liver was immediately dissected, rinsed in ice‐cold 0.9% sodium chloride solution, gently blotted to remove excess moisture, and immediately frozen in liquid nitrogen. All tissue samples were subsequently stored at −80°C until further analysis. To prepare enzyme extracts, cryopreserved liver tissue was subjected to homogenization in a 1.5% potassium chloride (KCl) solution using a 1:10 weight‐to‐volume ratio. The homogenate was then centrifuged at 9000 × g for 30 min at 4°C, and the resulting supernatant was carefully collected for downstream biochemical assays [46].

#### 2.6.2. Enzyme Assays and Lipid Peroxidation Analysis

The activities of several principal antioxidant enzymes were evaluated using spectrophotometric methods. The activity of superoxide dismutase (SOD) was determined by assessing its inhibitory effect on the photoreduction of NBT, according to the procedure described by Yazdanparast et al. [47]. Measurement of catalase (CAT) activity was performed by observing the rate at which hydrogen peroxide (H_2_O_2_) was decomposed at 240 nm, as reported by Takahara et al. [48]. The enzymatic activity of glutathione peroxidase (GPx) was examined by determining the NADPH oxidation rate, as outlined by Arun et al. [49]. Lipid peroxidation was further evaluated through the measurement of malondialdehyde (MDA) using the thiobarbituric acid (TBA) method, following the procedure established by Ohkawa et al. [50].

#### 2.6.3. Intestinal Sample Preparation and Digestive Enzyme Assays

Fish were subjected to a 24‐h feed deprivation period prior to sampling to ensure gut clearance and minimize the influence of recent feeding on digestive enzyme activity. Concurrently with liver sampling, the anterior intestine, including the pyloric ceca systematically dissected from each fish, with its contents delicately removed. The excised tissue was then cleansed using an ice‐chilled 0.9% sodium chloride solution. Intestinal samples were processed to extract crude enzymes by homogenization in a 50 mM Tris‐HCl buffer (pH 7.5) with a 1:5 tissue‐to‐buffer ratio (w/v). Following homogenization, the mixture was centrifuged at 10,000 × g for 15 min at 4°C, and the clarified supernatant was collected for analysis.

The activities of key digestive enzymes were determined spectrophotometrically. α‐Amylase enzymatic activity was quantified through starch hydrolysis, as per the methodology proposed by Bernfeld [51]. The activity of alkaline proteases was assessed through azocasein hydrolysis, following the method established by Chong et al. [52]. Lipase activity was assessed by quantifying the hydrolysis of p‐nitrophenyl myristate, following the method of Iijima et al. [53].

### 2.7. Gene Expression Analysis by Real‐Time Quantitative PCR (RT‐qPCR)

#### 2.7.1. RNA Isolation and cDNA Synthesis

Total RNA was extracted from liver tissues using a commercial column‐based kit following the manufacturer’s instructions. RNA integrity was verified by agarose gel electrophoresis, and concentration and purity were assessed spectrophotometrically. First‐strand cDNA was synthesized from 1 µg of total RNA and stored at −80°C until further analysis.

#### 2.7.2. RT‐qPCR

Gene expression of *hsp70-α* and *il-1β* was quantified using SYBR Green‐based RT‐qPCR. Reactions were performed in 10 µL volumes containing cDNA template, SYBR Green Master Mix, and gene‐specific primers (Table 3). β‐actin was used as the reference gene. The amplification protocol consisted of initial denaturation followed by 40 cycles of denaturation, annealing, and extension. Specificity of amplification was confirmed by melt curve analysis. Relative gene expression was calculated using the 2 ^(-ΔΔCT)^ method.

**Table 3:** The PCR fragment sizes associated with the primer pairs applied in this study for assessing gene expression in rainbow trout.

Primer name	Primer sequence (5’–3’)	Ta (°C)	Amplification efficiency (%)	Product size (Bp)	AN
β‐actin	F:CGAGACATCAAGGAGAAGC R:CCATACCGAGGAAGGAGG	64	95.38	181	XM_021595780
HSP70‐α	F:GGCTCAGCAAAGAGGATATT R:CTCCACGCTGCTCTTCATATT	64	97.74	167	XM_036964716
IL‐1β	F:TCTACCTGTCCTGCTCCAA R:GTCCGTGCTGATGAACCA	64	95.99	191	NM_001124347

*Note:* AN refers to the NCBI GenBank accession number corresponding to the rainbow trout reference gene sequence utilized in primer design.

### 2.8. Statistical Analysis

Data were tested for normality and homogeneity of variances using the Shapiro–Wilk and Levene’s tests. The effects of dietary supplementation, rearing temperature, sampling time, and their interactions were analyzed using three‐way ANOVA. When significant differences were detected, Tukey’s HSD test was applied for multiple comparisons. Statistical significance was set at *p*  < 0.05. Results are presented as mean ± SEM.

## 3. Results

### 3.1. Fish Performance (Growth, Feed Efficiency, and Proximate Analysis)

The performance and proximate composition of rainbow trout fed diets containing CoQ10 andO‐Se (Sel‐Plex) and reared at 15 and 20°C are summarized in Tables 4–6. Growth metrics, including WG, SGR, DGR, and TGC, were significantly influenced by both CoQ10 supplementation and water temperature (Table 4, *p*  < 0.05). Fish receiving the combined Sel‐Plex + CoQ10 diet at 15°C exhibited the highest WG (275.8), SGR (2.36), DGR (1.05), and TGC (0.18), whereas those fed the control diet at 20°C recorded the lowest performance (WG: 126.6; SGR: 1.46; DGR: 0.48; TGC: 0.08) (Table 4).

**Table 4:** Growth performance of fish fed diets containing organic selenium and coenzyme Q10 reared at 15 and 20°C temperatures.

Diets	WG	SGR	DGR	TGC
T_15_Se_0_Q_0_	235.98 ± 29.9^a^	2.16 ± 0.16^a^	0.89 ± 0.11^a^	0.16 ± 0.01^a^
T_15_Se_5_Q_0_	251.3 ± 1.5^a^	2.24 ± 0.01^a^	0.95 ± 0.01^a^	0.17 ± 0.01^a^
T_15_Se_0_Q_20_	268 ± 5.9^a^^∗^	2.33 ± 0.03^a^^∗^	1.02 ± 0.02^a^^∗^	0.18 ± 0.01^a^^∗^
T_15_Se_5_Q_20_	275.8 ± 2.3^a^^∗^	2.36 ± 0.01^a^^∗^	1.05 ± 0.01^a^^∗^	0.18 ± 0.01^a^^∗^
T_20_Se_0_Q_0_	126.6 ± 15.6^b^	1.46 ± 0.12^b^	0.48 ± 0.06^b^	0.08 ± 0.01^b^
T_20_Se_5_Q_0_	150.5 ± 6^b^	1.39 ± 0.04^b^	0.57 ± 0.02^b^	0.09 ± 0.01^b^
T_20_Se_0_Q_20_	163.3 ± 5.3^b^^∗^	1.73 ± 0.04^b^^∗^	0.62 ± 0.02^b^^∗^	0.09 ± 0.01^b^^∗^
T_20_Se_5_Q_20_	161 ± 3^b^^∗^	1.71 ± 0.02^b^^∗^	0.61 ± 0.01^b^^∗^	0.09 ± 0.01^b^^∗^

*Note:* Results are expressed as mean ± SE (*n* = 3). Values within a column marked with distinct superscripts (a, b) indicate significant differences (*p*  < 0.05). An asterisk ( ^∗^) indicates significant effects of temperature on growth performance.

Abbreviations: DGR, daily growth rate (g day^−1^); SGR, specific growth rate (% day^−1^); TGC (%), thermal growth coefficient; WG, weight gain (g).

**Table 5:** Feed efficiency of fish fed diets containing organic selenium and coenzyme Q10 reared at 15 and 20°C temperatures.

Diets	FCR	PER	LER	PPV	LPV
T_15_Se_0_Q_0_	1.23 ± 0.15^c^	5.8 ± 0.05^a^	20.67 ± 0.41^a^	0.44 ± 0.04^a^	0.17 ± 0.02^a^
T_15_Se_5_Q_0_	1.26 ± 0.01^c^	5.83 ± 0.01^a^	21.5 ± 0.72^a^	0.47 ± 0.01^a^^∗^	0.18 ± 0.01^a^^∗^
T_15_Se_0_Q_20_	1.16 ± 0.03^c^	5.91 ± 0.03^a^^∗^	22.23 ± 0.8^a^	0.5 ± 0.02^a^	0.19 ± 0.01^a^
T_15_Se_5_Q_20_	1.15 ± 0.01^c^	5.87 ± 0.03^a^^∗^	21.5 ± 1.1^a^	0.31 ± 0.02^a^^∗^	0.12 ± 0.01^a^^∗^
T_20_Se_0_Q_0_	1.97 ± 0.05^a^	5.37 ± 0.22^b^	20.05 ± 0.4^a^	0.34 ± 0.03^b^	0.12 ± 0.01^b^
T_20_Se_5_Q_0_	1.73 ± 0.06^b^	5.44 ± 0.05^b^	20.09 ± 0.4^a^	0.38 ± 0.02^b^^∗^	0.14 ± 0.01^b^^∗^
T_20_Se_0_Q_20_	1.67 ± 0.01^b^^∗^	5.63 ± 0.08^b^^∗^	21.16 ± 1.3^a^	0.37 ± 0.01^b^	0.14 ± 0.01^b^
T_20_Se_5_Q_20_	1.72 ± 0.01^b^^∗^	5.7 ± 0.09^b^^∗^	20.19 ± 0.21^a^	0.36 ± 0.01^b^^∗^	0.14 ± 0.01^b^^∗^

*Note:* Results are expressed as mean ± SE (*n* = 3). Values within a column marked with distinct superscripts (a, b) indicate significant differences (*p*  < 0.05). An asterisk ( ^∗^) indicates significant effects of temperature factor on feed efficiency.

Abbreviations: FCR, feed conversion ratio; FER, feed efficiency ratio; LER, lipid efficiency ratio; LPV (%), lipid production value; PPV (%), protein production value.

**Table 6:** Proximate composition (%) of fish fed diets containing organic selenium and coenzyme Q10 reared at 15 and 20°C temperatures.

Diets	Moisture	Protein	Fat	Ash
T_15_Se_0_Q_0_	65.80 ± 0.60	15.69 ± 0.04	4.93 ± 0.06^a^	14.24 ± 0.71
T_15_Se_5_Q_0_	65.10 ± 0.10	15.72 ± 0.02	4.79 ± 0.11^a^	14.83 ± 0.25
T_15_Se_0_Q_20_	65.70 ± 0.50	15.62 ± 0.09	4.67 ± 0.11^a^	14.73 ± 0.36
T_15_Se_5_Q_20_	66.10 ± 0.10	15.72 ± 0.06	4.79 ± 0.18^a^	14.57 ± 0.42
T_20_Se_0_Q_0_	65.90 ± 0.60	15.73 ± 0.23	5.04 ± 0.06^b^	14.26 ± 0.24
T_20_Se_5_Q_0_	65.20 ± 0.50	15.88 ± 0.04	5.04 ± 0.06^b^	14.61 ± 0.05
T_20_Se_0_Q_20_	65.15 ± 0.80	15.61 ± 0.19	4.89 ± 0.18^b^	15.01 ± 0.02
T_20_Se_5_Q_20_	65.10 ± 0.10	15.45 ± 0.15	5.02 ± 0.03^b^	15.03 ± 0.01

*Note:* Results are expressed as mean ± SE (*n* = 3). Values within a column marked with distinct superscripts (a, b) indicate significant differences (*p*  < 0.05).

Feed utilization indices were similarly affected (Table 5). The highest FCR was observed in fish on the control diet at 20°C (1.97), whereas the lowest FCR occurred in fish fed the combined CoQ10 + Sel‐Plex diet at 15°C (1.15) (*p*  < 0.05). (PER ranged from 5.91 in the CoQ10 15°C group to 5.37 in the control 20°C group (*p*  < 0.05). Similarly, protein production value (PPV) and lipid production value (LPV) were significantly affected by diet and temperature, with the highest PPV (0.50) and LPV (0.19) observed in the CoQ10 15°C group, and the lowest PPV (0.31) and LPV (0.12) recorded in the CoQ10 + Sel‐Plex 15°C group (*p*  < 0.05).

Whole‐body composition was affected primarily by fat content (Table 6). Fish reared at 20°C without dietary supplementation exhibited the highest fat content (5.04), while the lowest fat content (4.67) was found in the CoQ10 15°C group (*p*  < 0.05). No significant differences were observed in moisture, protein, or ash contents among the experimental groups (*p*  > 0.05).

### 3.2. Serum Biochemistry

**Figure 1:**
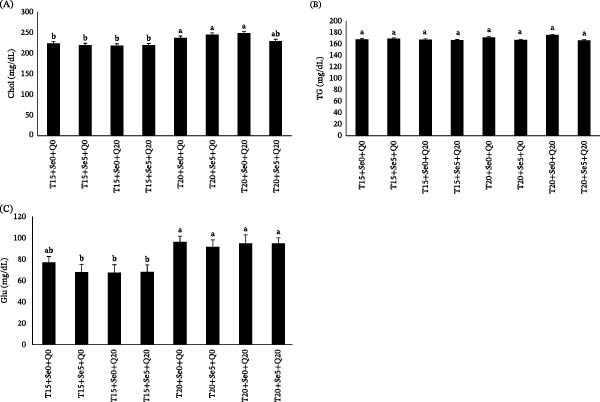
Serum Chol (A), TG (B), and Glu (C) in fish fed diets containing organic selenium and coenzyme Q10 reared at 15 and 20°C temperatures. Values (mean ± SE) are presented, with columns labeled by different lowercase letters representing statistically significant differences at *p* < 0.05.

The effects of experimental diets on serum biochemical indices are presented in Figure 1. All groups reared at 20°C exhibited significantly higher serum Chol content, except for the fish fed a diet supplemented by CoQ10 + Sel‐Plex (Figure 1A). TG levels remained unchanged across the experimental diets and rearing temperature conditions (Figure 1B). Fish grown at the elevated water temperature of 20°C had significantly higher Glu levels compared to those grown at normal water temperature and fed antioxidant‐supplemented diets (Figure 1C).

### 3.3. Innate Immune Indices

**Figure 2:**
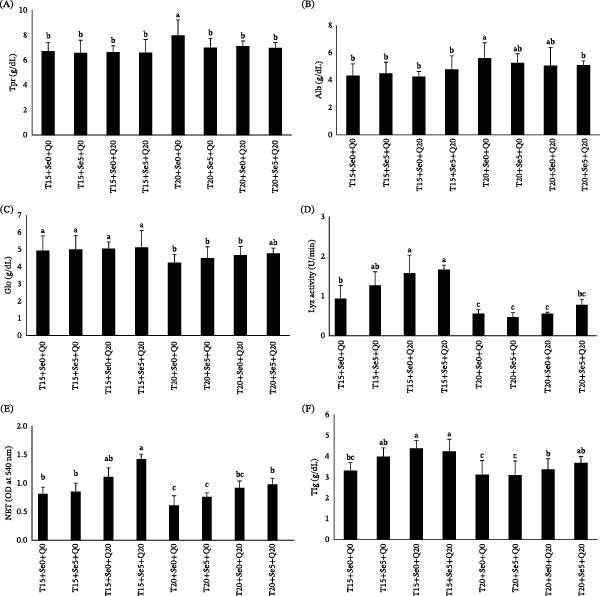
Serum Tpr (A), Alb (B), Glo (C), Lyz activity (D), respiratory burst activity (E), and TIg (F) in fish fed diets containing organic selenium and coenzyme Q10 reared at 15 and 20°C temperatures. Values (mean ± SE) are presented, with columns labeled by different lowercase letters representing statistically significant differences at *p* < 0.05.

Fish grown at 20°C exhibited significantly higher serum concentrations of Tpr and Alb (Figure 2A, B). Conversely, all experimental groups exposed to 20°C exhibited significantly lower serum Glo levels compared with those maintained at the standard temperature of 15°C, except for fish that received dietary supplementation with both antioxidants (Sel‐Plex + CoQ10) (Figure 2C).

Diets, water temperatures, and their interaction significantly influenced Lyz activity. Fish reared at normal water temperature significantly showed higher Lyz activity compared to those grown at elevated temperatures. The interaction between dietary supplements and rearing water temperature had a notable impact on serum Lyz activity; specifically, the Lyz activity of fish grown at 20°C and fed diets cosupplemented with antioxidants (T20, Se5+Q20) was comparable to that of fish reared at normal water temperature and fed a diet without supplements. Conversely, feeding fish at 15°C with diets containing CoQ10 or a combination of Se and CoQ10 resulted in the highest Lyz activity (Figure 2D).

Exposure to standard water temperatures combined with a diet fortified with Sel‐Plex and CoQ10 resulted in the greatest respiratory burst activity in fish. In contrast, fish kept at an elevated temperature showed a significant reduction in respiratory burst activity. However, the administration of Sel‐Plex and CoQ10 was able to enhance respiratory burst activity to levels comparable to those of the control fish maintained at 15°C (Figure 2E).

Fish reared at normal water temperature that were fed a diet supplemented with any form of antioxidants exhibited significantly elevated levels of Ig compared to the control group. A parallel trend was obtained in fish housed at elevated temperature, where those nourished with CoQ10 or a combination of both antioxidants displayed plasma Ig levels comparable to those in control fish (Figure 2F).

### 3.4. Hepatic Antioxidant Status

**Figure 3:**
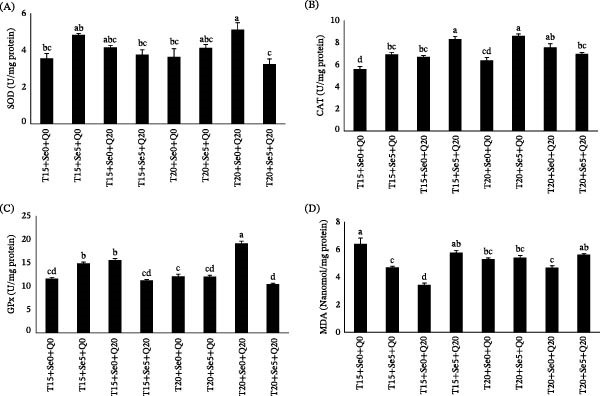
Antioxidant enzymes activity, (A) superoxide dismutase (SOD), (B) catalase (CAT), (C) glutathione peroxidase (GPx), as well as (D) malondialdehyde content (MDA), of fish fed diets containing organic selenium and coenzyme Q10 reared at 15 and 20°C temperatures. Values (mean ± SE) in columns annotated with distinct lowercase letters (a, b, c, d) are significantly different at *p* < 0.05.

The effects of dietary CoQ10 and O‐Se (Sel‐Plex) on the hepatic antioxidant status of rainbow trout reared at two different temperatures are presented in Figure 3.

The experimental treatments significantly affected the activities of all measured antioxidant enzymes (*p*  < 0.05). SOD activity reached its maximum in the group receiving CoQ10 at 20°C, whereas the lowest activity occurred in fish fed Sel‐Plex at 15°C and in those receiving the combined supplementation at 20°C. For CAT, the highest activity was observed in the Sel‐Plex/20°C group, with the control group exhibiting the lowest activity. Similarly, GPx activity peaked in the CoQ10/20°C group and was lowest in the CoQ10 + Sel‐Plex/20°C group. Hepatic levels of MDA, indicative of lipid peroxidation, were found to be higher in the control group than in most other treatments (*p*  < 0.05), whereas the CoQ10/15°C group displayed the lowest level.

### 3.5. Digestive Enzymes Activity

**Figure 4:**
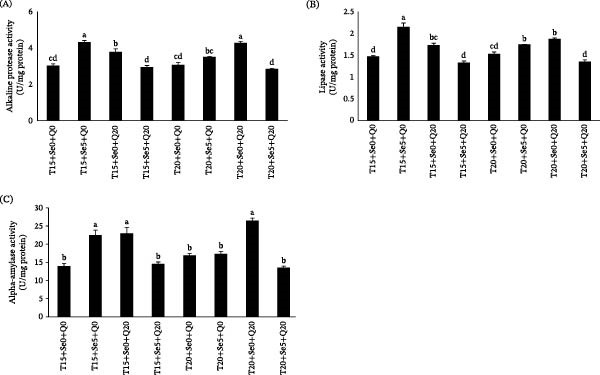
Digestive enzymes activity, alkaline protease (A), lipase (B), and alpha‐amylase (C) of fish fed diets containing organic selenium and coenzyme Q10 reared at 15 and 20°C temperatures. Values (mean ± SE) in columns annotated with distinct lowercase letters (a, b, c, d) are significantly different at *p* < 0.05.

The effects of dietary enrichment with CoQ10 and Sel‐Plex and the activity of digestive enzymes in fish maintained at 15 and 20°C is demonstrated in Figure 4. The Sel‐Plex/T15°C, CoQ10/T15°C, and CoQ10/T20°C groups exhibited significantly altered alkaline protease activity compared to the control group (*p*  < 0.05, Figure 4A). Enzymatic activity peaked among fish supplemented with Sel‐Plex at 15°C, whereas in contrast, the minimum activity was noted in fish provided a diet containing both CoQ10 and Sel‐Plex at 20°C. A notable variation in lipase‐specific activity was observed between the control and all experimental treatments (*p*  < 0.05, Figure 4B). The highest lipase activity was recorded in the Sel‐Plex/T15°C group, whereas the lowest activity was detected in the CoQ10 + Sel‐Plex/T15°C group. Conversely, α‐amylase activity in the control group was significantly higher than in the Sel‐Plex/T15°C, Sel‐Plex/T20°C, and CoQ10/T20°C groups (*p*  < 0.05, Figure 4C).

### 3.6. Expression Patterns of *il-1β* and *hsp70*‐*α*

**Figure 5:**
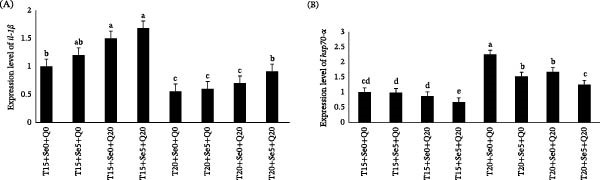
Effect of thermal acclimation and diets supplemented with antioxidants on the gene expression levels of *il-1β* (A) and *hsp70-α* (B) in rainbow trout fingerlings. Different lowercase letters indicate statistically significant differences (*p* < 0.05) among treatments. The control group was designated as the reference with a baseline value of 1, and gene expression in the remaining groups is presented relative to this baseline.

Fish exposed to baseline temperature conditions exhibited greater *il-1β* expression in comparison to those maintained at elevated temperatures. However, the addition of supplements enhanced *il-1β* expression in fish fed diets containing CoQ10 or Se alone and CoQ10 + Se at optimal temperature and also fish fed diets containing CoQ10 + Se at elevated temperature (*p*  < 0.05, Figure 5A). The peak expression of *il-1β* was observed at 15°C in fish that received both supplements, while fish in elevated water temperatures that were fed a basal diet (without supplements) or supplements alone exhibited significantly lower *il-1β* expression (*p*  < 0.05).

The expression pattern of *hsp70-α* was somewhat contrary to that of *il-1β*. The expression of this gene exhibited a slight decreasing trend with the addition of dietary supplements (Figure 5B). More importantly, *hsp70-α* expression levels were significantly different between cold and warm water conditions overall. Fish exposed to elevated temperatures exhibited upregulated *hsp70-α* expression, whereas antioxidant supplementation attenuated this gene expression. Although fish in warm water fed a basal diet exhibited the highest expression level of *hsp70-α*, groups that received Sel‐plex and/or Co‐Q10 had lower expression levels of *hsp70-α*. Furthermore, *hsp70-α* expression in the group administered the combined antioxidants (T20+Se5+Q20) remained comparable to that of the control group, which was kept at 15°C and provided a basal diet. This suggests that the coadministration of antioxidants may mitigate the physiological effects of thermal stress in fish reared under higher temperature conditions.

## 4. Discussion

Rainbow trout grow optimally at 8–15°C, with acclimation, strain, and habitat influencing thermal preferences [54, 55]. In the present study, fish reared at 15°C exhibited higher growth and feed efficiency than those at 20°C, consistent with previous findings showing reduced growth and feed utilization at supra‐optimal temperatures [56–59, 60]. These results indicate that elevated water temperature can impair nutrient utilization and limit growth in rainbow trout. Elevated water temperatures markedly influence energy allocation and nutrient metabolism in fish, especially in salmonids [61]. As ectothermic organisms, their metabolic rate rises with temperature, directing more metabolizable energy toward maintenance and physiological homeostasis, which reduces the energy available for growth [6, 34]. Protein metabolism is particularly affected; thermal stress can compromise digestive enzyme efficiency and protein digestibility while increasing protein turnover to support stress‐response mechanisms, such as heat shock protein synthesis [62, 63]. Lipid metabolism is also sensitive, with elevated temperatures impacting digestibility, synthesis, and membrane composition, potentially raising oxidative stress and energy costs for repair [64]. Consequently, reduced growth under high‐temperature conditions reflects the reallocation of energy toward maintenance and stress mitigation rather than feed intake alone, highlighting the importance of targeted nutritional strategies to support growth and feed efficiency under thermal stress.

Whole‐body fat content increased in fish reared at elevated temperatures, contrasting with previous reports of decreased fat under heat stress [65]. Growth performance was improved in rainbow trout fed diets supplemented with CoQ10 and O‐Se at 15°C. Similar benefits of CoQ10 and O‐Se supplementation on growth have been reported in Nile tilapia and African catfish [23, 33]. In some studies, antioxidant supplementation showed no significant effect, likely due to differences in dosage or experimental conditions [36]. The observed growth enhancement may be linked to improved intestinal health and the anti‐inflammatory effects of CoQ10 [66], while Se in organic form consistently supports growth and physiological performance in fish [67–69].

In this study, Sel‐Plex, an O‐Se source derived from selenized yeast, was used as a dietary supplement. This form of Se is rich in selenoproteins, which are known to enhance antioxidant defenses and regulate key metabolic processes [70]. Among these selenoproteins, type 1 iodothyronine 5^′^‐deiodinase plays a crucial role in thyroid hormone metabolism, thereby supporting growth and preventing metabolic disturbances [71]. Under stress conditions, such as elevated water temperatures, dietary Se has been demonstrated to improve antioxidant capacity, reduce oxidative stress, and maintain physiological homeostasis in fish [24, 32, 72]. Thus, the enhanced growth performance observed in fish fed diets supplemented with O‐Se can be attributed to Se’s ability to mitigate stress‐induced metabolic constraints and to support efficient nutrient utilization under the experimental conditions [73].

Blood biochemical indices are reliable indicators of stress‐induced physiological changes in fish [74]. In the present study, elevated water temperatures significantly increased serum Chol and Glu, reflecting physiological stress [75], while total protein and albumin also rose, likely indicating tissue repair and osmoregulatory adjustments [60, 76]. Conversely, serum globulin, a key component of Igs, decreased under thermal stress, suggesting compromised immunity. Dietary supplementation with CoQ10 and O‐Se mitigated these adverse effects, improving serum protein profiles and supporting immune function in fish exposed to elevated temperatures [60, 77].

Elevated water temperature significantly reduced serum Lyz activity, indicating suppression of innate immune function under thermal stress. Dietary antioxidant supplementation enhanced Lyz activity regardless of temperature, and the combined administration of CoQ10 and O‐Se restored Lyz levels in heat‐stressed fish to values comparable with those of fish maintained at optimal temperature. Given the essential role of Lyz in innate immunity, these findings demonstrate that both environmental conditions and nutritional interventions modulate immune resilience in fish under stress [78]. The results are consistent with previous observations reporting enhanced serum Lyz activity following Se and *Pediococcus acidilactici* supplementation in rainbow trout [79].

Igs, as key mediators of adaptive immunity, were significantly enhanced by dietary supplementation with CoQ10 and O‐Se under both normal and elevated temperature conditions. These findings align with previous evidence demonstrating that CoQ10 supplementation improves immune responses in Nile tilapia (*Oreochromis niloticus*) [75] and that combined antioxidant supplementation, including nano‐Se (Nano‐Se), CoQ10, and vitamin E, enhances stress tolerance in rainbow trout [36]. The increased serum Ig and Lyz levels may be attributed to antioxidant‐induced stimulation of leukocyte and neutrophil proliferation [80].

Phagocytosis, a fundamental innate defense mechanism mediated through respiratory burst activity, was significantly enhanced in fish supplemented with O‐Se and CoQ10 under both normal and elevated temperature conditions. This enhancement reflects increased production of reactive oxygen species (ROS) involved in pathogen elimination [81]. The results are consistent with Dawood et al. [82], who reported elevated respiratory burst activity in red sea bream (*Pagrus major*) fed Se‐NP–supplemented diets.

Oxidative stress results from an imbalance between ROS generation and antioxidant defenses. CAT and GPx play central roles in ROS detoxification [83]. In the present study, dietary CoQ10 and O‐Se enhanced hepatic antioxidant enzyme activities in rainbow trout, consistent with previous reports documenting similar stress‐mitigating effects in various fish species [22, 24, 33, 36, 75].

CoQ10 inhibits superoxide generation and is inversely associated with hydrogen peroxide levels [84], while Se supports antioxidant defense as a structural component of GPx, thereby reducing ROS‐induced oxidative stress [85]. Decreased tissue MDA reflects enhanced antioxidant capacity and protection against lipid peroxidation [86]. In this study, antioxidant‐enriched diets lowered MDA levels compared to controls, consistent with findings in rainbow trout supplemented with Nano‐Se, vitamin C, and vitamin E [87] and in European eel fed CoQ10 [24].

Water temperature markedly influences digestive physiology and nutrient utilization in fish by altering feeding behavior, gastrointestinal transit, membrane fluidity, and enzyme secretion [88]. Thermal stress accelerates metabolism and ROS production, potentially impairing intestinal and pancreatic function and reducing growth performance, particularly in cold‐water species reared above their optimal range [13, 14]. In the present study, rainbow trout maintained at 20°C exhibited reduced growth; however, dietary supplementation with CoQ10 or O‐Se enhanced digestive enzyme activities. Improved enzyme activity is generally linked to better nutrient digestibility [89], suggesting that antioxidant supplementation preserved digestive functionality under heat stress. The beneficial effects may relate to the complementary roles of CoQ10 in mitochondrial bioenergetics and ATP production [21, 84, 90, 91] and Se in redox regulation via selenoproteins such as GPx [92]. Similar enhancements in digestive enzyme activity following CoQ10 or Se supplementation have been reported in teleosts [75, 93–96], with synergistic effects also documented in trout under stress conditions [36]. Collectively, these findings suggest that improved redox balance and mitochondrial protection, rather than direct stimulation of enzyme synthesis, underpin the enhanced digestive performance observed under thermal challenge.

Significant variation in *il-1β* expression was observed across treatments, indicating that both temperature and dietary supplementation modulate immune‐related gene regulation. Compared with the basal diet, supplemented groups exhibited elevated *il-1β* expression, suggesting that targeted nutritional interventions can mitigate heat stress–induced immune suppression and enhance stress tolerance in rainbow trout. Temperature‐dependent changes further demonstrate the adaptive capacity of trout to modulate immune responses under thermal challenge, particularly in the presence of antioxidants [97].

This study evaluated *hsp70-α* expression in rainbow trout under different dietary and thermal conditions and observed significant modulation by both factors. Elevated temperature induced upregulation of *hsp70-α*, whereas combined antioxidant supplementation attenuated this response, highlighting the influence of dietary components on stress‐regulatory mechanisms [98]. Although increased *hsp* expression enhances thermal tolerance [97], its sustained activation imposes substantial bioenergetic costs [99]. Antioxidant supplementation likely reduced the need for excessive *hsp* overexpression by limiting ROS‐induced protein damage, thereby lowering energy expenditure [100]. These findings demonstrate the interactive effects of diet and temperature on molecular stress responses and adaptive capacity in rainbow trout.

## 5. Conclusion

Elevated water temperature (20°C) negatively affected growth performance, digestive activity, antioxidant capacity, and immune responses in rainbow trout, as reflected by impaired physiological indices and upregulation of stress‐related genes (HSP70‐α and IL‐1β). Dietary supplementation with CoQ10 and O‐Se partially alleviated these adverse effects, with the combined treatment showing the most pronounced improvements in growth, antioxidant status, and innate immunity. These findings suggest that antioxidant‐based nutritional strategies may enhance thermal resilience in rainbow trout under moderate heat stress. However, the study was conducted under controlled conditions using a single temperature and fixed supplementation levels. Further research is required to determine optimal dosages, long‐term effects, and applicability under commercial farming conditions.

## Author Contributions

Mohammad Hossein Dadfar has conducted the research, written early draft of the MS, and also collected the data. Ahmad Imani was involved in grant acquisition, experimental design, data analyses and interpretation, and manuscript reviewing and editing. Arya Vazirzadeh has actively contributed to grant acquisition, data collection, and manuscript reviewing and editing. Kourosh Sarvi Moghanlou was also involved in data interpretation, manuscript reviewing and editing. Faezeh Jamali was involved in the investigation and collection of data.

## Funding

The research was supported by the Research Council of Urmia University.

## Disclosure

All authors have reviewed the manuscript.

## Ethics Statement

The present experiment was conducted under standard laboratory conditions in terms of experimental setup, animal care and use and approved by the Veterinary Ethics Committee of Urmia University (IR‐UU‐AEC‐3/71).

## Consent

The authors have nothing to report.

## Conflicts of Interest

The authors declare no conflicts of interest.

## Data Availability

All data generated or analyzed during this study were presented in this published article.
